# Interspecies Chromosome Mapping in Caprimulgiformes, Piciformes, Suliformes, and Trogoniformes (Aves): Cytogenomic Insight into Microchromosome Organization and Karyotype Evolution in Birds

**DOI:** 10.3390/cells10040826

**Published:** 2021-04-07

**Authors:** Rafael Kretschmer, Marcelo Santos de Souza, Ivanete de Oliveira Furo, Michael N. Romanov, Ricardo José Gunski, Analía del Valle Garnero, Thales Renato Ochotorena de Freitas, Edivaldo Herculano Corrêa de Oliveira, Rebecca E. O’Connor, Darren K. Griffin

**Affiliations:** 1 School of Biosciences, University of Kent, Canterbury CT2 7NJ, UK; rafa.kretschmer@hotmail.com (R.K.); m.romanov@kent.ac.uk (M.N.R.); rebeckyoc@gmail.com (R.E.O.); 2 Departamento de Genética, Universidade Federal do Rio Grande do Sul, Porto Alegre, 91509-900 Rio Grande do Sul, Brazil; thales.freitas@ufrgs.br; 3 Laboratório de Diversidade Genética Animal, Universidade Federal do Pampa, São Gabriel, 97300-162 Rio Grande do Sul, Brazil; marcelodesouzabio@gmail.com (M.S.d.S.); ricardogunski@unipampa.edu.br (R.J.G.); analiagarnero@unipampa.edu.br (A.d.V.G.); 4 Laboratório de Reprodução Animal, LABRAC, Universidade Federal Rural da Amazônia, UFRA, Parauapebas, 68515-000 Pará, Brazil; ivanetefuro100@gmail.com; 5 Laboratório de Cultura de Tecidos e Citogenética, SAMAM, Instituto Evandro Chagas, Ananindeua, 67030-000 Pará, Brazil; ehco@ufpa.br; 6 Instituto de Ciências Exatas e Naturais, Universidade Federal do Pará, Belém, 66075-110 Pará, Brazil

**Keywords:** avian cytogenomics, evolution, genome organization, FISH, chromosomal rearrangements

## Abstract

Interchromosomal rearrangements involving microchromosomes are rare events in birds. To date, they have been found mostly in Psittaciformes, Falconiformes, and Cuculiformes, although only a few orders have been analyzed. Hence, cytogenomic studies focusing on microchromosomes in species belonging to different bird orders are essential to shed more light on the avian chromosome and karyotype evolution. Based on this, we performed a comparative chromosome mapping for chicken microchromosomes 10 to 28 using interspecies BAC-based FISH hybridization in five species, representing four Neoaves orders (Caprimulgiformes, Piciformes, Suliformes, and Trogoniformes). Our results suggest that the ancestral microchromosomal syntenies are conserved in *Pteroglossus inscriptus* (Piciformes), *Ramphastos tucanus tucanus* (Piciformes), and *Trogon surrucura surrucura* (Trogoniformes). On the other hand, chromosome reorganization in *Phalacrocorax brasilianus* (Suliformes) and *Hydropsalis torquata* (Caprimulgiformes) included fusions involving both macro- and microchromosomes. Fissions in macrochromosomes were observed in *P*. *brasilianus* and *H*. *torquata*. Relevant hypothetical Neognathae and Neoaves ancestral karyotypes were reconstructed to trace these rearrangements. We found no interchromosomal rearrangement involving microchromosomes to be shared between avian orders where rearrangements were detected. Our findings suggest that convergent evolution involving microchromosomal change is a rare event in birds and may be appropriate in cytotaxonomic inferences in orders where these rearrangements occurred.

## 1. Introduction

Birds (class Aves) are the most diverse lineage of extant tetrapod vertebrates, comprising 10,806 extant species, divided into 40 extant avian orders [1]. Despite the extraordinary diversity in morphology, ecology and behavior [2], a high proportion of species analyzed so far showed karyotypes composed of about 80 chromosomes, consisting of a few large macrochromosomes (~10) and numerous microchromosomes (~30) [3,4,5]. This cytogenomic structure is mostly conserved since the Archelosaur common ancestor and is thought to be a feature of non-avian dinosaurs [6]. Some exceptions to the typical avian karyotype are seen within the superorder Neoaves from the infraclass Neognathae, including the orders Falconiformes [7], Psittaciformes [8], and Ciconiiformes [9], which have reduced diploid numbers, and Piciformes having higher diploid numbers [10,11]. The decrease or increase of chromosome number can result from fusion and fission events, respectively [12]. According to Imai et al. [13] and their “minimum-interaction hypothesis”, the karyotype evolution tends to increase the diploid number and the number of acrocentric chromosomes by centric fissions, minimizing the risk of deleterious rearrangements. In addition, these authors suggested that the relative probability of reciprocal translocations (i.e., centric fusion) declines with increases in chromosome number and in nuclear volume.

A lower number of microchromosomes in avian species with reduced diploid numbers is the most prominent karyotypic difference as compared to species with greater diploid numbers [4]. Although the analyses of nucleotide substitution patterns in two representatives of the order Galliformes from the superorder Galloanserae, chicken (*Gallus gallus*, GGA) and Turkey (*Meleagris gallopavo*) [14], have revealed a higher rate of sequence evolution on microchromosomes as compared to macrochromosomes, these tiny elements appear to be highly conserved syntenically and not prone to breakage [15]. On the other hand, using cross-species fluorescent in situ hybridization (FISH), chromosome fissions have been reported for almost all the avian macrochromosomes (except GGA8 and 10) [3,4,5,16], most of them in the first five autosomal pairs (GGA1–5) [4,5]. The breakpoint regions involved in these chromosomal rearrangements are usually associated with genomic features, including transposable elements and conserved noncoding elements. It has been suggested that they are reused in avian chromosome evolution [17,18].

Avian karyotypes have been investigated over the last decades by whole chromosome painting using different probes sets [3,4]. These analyses have been an important tool to detect chromosomal similarities and differences between species and changes in each lineage since they diverged from common ancestors, which can also be virtually reconstructed using software algorithms (e.g., [6,19,20,21,22,23]). However, this approach has been applied to less than 1% of species, and so many avian orders have no information concerning chromosomal homology based on molecular cytogenetics [4,5]. Hence, our knowledge about chromosome organization in birds remains somewhat patchy, especially because most of the studies have included only probes corresponding to ancestral macrochromosomes (homology to GGA 1–9) [3,4].

Recently, bacterial artificial chromosome (BAC) probes from the genomic libraries of chicken and zebra finch (*Taeniopygia guttata*, Passeriformes, Neoaves) have been applied successfully across multiple avian species totaling chromosome maps for 36 species from 12 different orders [15,16,24,25,26,27,28,29,30]. Results from these studies suggested evolutionary stability in avian microchromosome organization, except in Falconiformes, Psittaciformes, and Cuculiformes species, in which microchromosomal fusions were found [15,16,24,25,27,28], demonstrating the usefulness of microchromosome BAC probes to provide a more extensive analysis of chromosomal evolution in birds.

In this regard, our research objective was to expand our cytogenomic understanding of microchromosome organization in birds using five species distributed in four different Neoaves orders, namely: scissor-tailed nightjar (*Hydropsalis torquata*, Caprimulgiformes), neotropic cormorant (*Phalacrocorax brasilianus*, Suliformes), lettered aracari (*Pteroglossus inscriptus*, Piciformes), red-billed toucan (*Ramphastos tucanus tucanus*, Piciformes), and surucua trogon (*Trogon surrucura surrucura*, Trogoniformes). We aimed at generating interspecies chromosome maps to verify if, during their evolutionary histories, different avian lineages underwent lineage-specific chromosomal rearrangements involving the microchromosomes. Considering that the Z sex chromosome is the largest chromosome in the karyotype of Piciformes species, we also examined a hypothesis that microchromosome fusions contributed to the enlargement of this chromosome. These hypotheses were tested by applying two BAC probes per microchromosome, derived from chicken or zebra finch, for microchromosomes GGA10–28 (except GGA16). In addition, BAC probes for macrochromosomes were applied to *H*. *torquata* and *P*. *brasilianus*, for which there is no chromosome painting data in species from these orders. Ultimately, presumed Neognathae and Neoaves ancestral karyotypes were reconstructed *in silico* to trace chromosomal changes in the evolutionary lineages of the five birds studied.

## 2. Materials and Methods

### 2.1. Specimens Analyzed and Chromosome Preparation

Skin biopsies or feather pulp samples were collected from one individual per species: from animals captured in their natural environment (*H. torquata* and *T. s. surrucura*) or from *ex situ* individuals (*P. brasilianus*, *P. inscriptus*, and *R. t. tucanus*) (Table 1). We performed fibroblast cell culture to obtain chromosome preparations. Cells were cultured in flasks (25 cm^2^) with DMEM cell culture media (Dulbecco’s Modified Eagle’s medium, Sigma-Aldrich, MO, USA), supplemented with 20% fetal bovine serum (GIBCO/Thermo Fisher Scientific, USA), 1% penicillin (10,000 units/mL)–streptomycin (10,000 μg/mL) (Sigma-Aldrich, St. Louis, MO, USA) and incubated at 37 °C [31]. Metaphase chromosomes were obtained according to standard procedures involving colcemid exposure (1 h, 37 °C), hypotonic treatment (0.075 M KCl, 15 min, 37 °C) and fixation with methanol:acetic acid (3:1).

### 2.2. Diploid Number and Karyotype Description

At least 20 metaphase spreads conventionally stained (Giemsa 5%) were analyzed to determine the diploid chromosome number and chromosomal morphologies for all studied species. Chromosomes were numbered consecutively based on their size and centromere position [33]. We performed a detailed karyotype description for *P*. *brasilianus* because this is the first cytogenetic analysis in this species.

### 2.3. Cross-Species FISH-Based Chromosome Maps

For the purpose of interspecies chromosome mapping, two BAC probes, selected from the genomic library of chicken or zebra finch and positioned as close as possible to the end of each microchromosome arms, were chosen for microchromosomes GGA10–28 (except GGA16) and applied to metaphases of *P*. *brasilianus*, *H*. *torquata*, *P*. *inscriptus*, *R*. *t*. *tucanus*, and *T*. *s*. *surrucura* (Table S1). The GGA16 and 29–38 were not tested here because there are no BAC probes available for these chromosomes. Two BAC probes per macrochromosome corresponding to GGA1–9 were also applied to metaphases of *P*. *brasilianus* and *H*. *torquata* (Table S1) since there were no prior studies of molecular cytogenetics in these species. Although we used BAC probes from chicken and zebra finch libraries, our results were compared with the chicken. Most of the BAC probes used were from the chicken. However, for some chromosomes, the chicken BACs did not work successfully in all bird species [24]; in these cases, we used BAC probes from the zebra finch. The BAC clone isolation, amplification, labeling and hybridization were performed following the O’Connor et al. [15] procedure. The FISH results were confirmed by analyzing at least 10 metaphase preparations per experiment.

### 2.4. Reconstruction of the Neognathae/Neoaves Ancestral Karyotypes

Datasets resulted from cross-species FISH-based mapping were used as input files for the software-assisted reconstruction of the hypothetical Neognathae and Neoaves ancestral karyotypes using the maximum-likelihood algorithm. For this purpose, we employed the Maximum Likelihood for Gene Order Analysis (MLGO) webserver [34]. The MLGO reconstruction algorithm built up a Neoaves ancestor (NAA) using the maximum-likelihood scenario for a certain number of common BACs among the five avian species studied plus the chicken reference karyotype. Being quite flexible, the algorithm can handle mixed datasets with missing/failed hybridization information for chromosome location of a particular BAC in a single species. For macrochromosome datasets in *P*. *brasilianus*, *H*. *torquata* and chicken, information about physical positions/orders of BACs relative to each other, p/q arms and centromeres was taken into account, enabling to reconstruct ancestral macrochromosomes in more detail.

Finally, the obtained interspecies FISH hybridization datasets were amended with information available from other relevant studies for six more species, including five other Neognathae/Neoaves representatives: smooth-billed ani (*Crotophaga ani*, Cuculiformes) [27], budgerigar (*Melopsittacus undulatus*, Psittaciformes) [16], saker falcon (*Falco cherrug*, Falconiformes) [16], peregrine falcon (*Falco peregrinus*, Falconiformes) [16,25], pigeon (*Columba livia*, Columbiformes) [23,35], and one Palaeognathae species, ostrich (*Struthio camelus*, Struthioniformes) [16]. This expanded dataset for 12 birds was employed for reconstructing *in silico* the Neognathae/Neoaves ancestral karyotypes using information about positions/orders of BACs on macrochromosomes, as well.

To run the MLGO web tool, a phylogenetic tree for the 12 species was recreated from a comprehensive avian phylogeny described by Prum et al. [36]. The correct tree topology (in Newick format) was tested using the ETE v3 toolkit [37] (Figure S1). As we established in our previous reconstruction studies [6,19,20,23], MLGO outputs were, by inference and manually, curated and adjusted further to interpret and correct software-assisted reconstruction results using the most parsimonious explanation of the available data.

### 2.5. Microscope Analysis and Image Capturing

Images were captured under a 100× objective using an Olympus BX61 epifluorescence microscope with a cooled CCD camera and SmartCapture (Digital Scientific UK, Cambridge, UK) system. Final image processing was performed using Adobe Photoshop 7.0 (accessed on 22 March 2021).

### 2.6. Statistical Analysis

Data of microchromosome rearrangements obtained in our study and from the literature were analyzed using a heterogeneity chi-squared test followed by analyses of residuals using WinPEPI software [38]. A statistically significant positive residual indicates an excess of species with rearrangements in a particular microchromosome. Residuals’ *p*-values were adjusted for multiple tests using Benjamini and Hochberg’s [39] false discovery rate (FDR) approach, with *p* ≤ 0.05 being considered statistically significant.

## 3. Results

### 3.1. Karyotype Description

**Figure 1:**
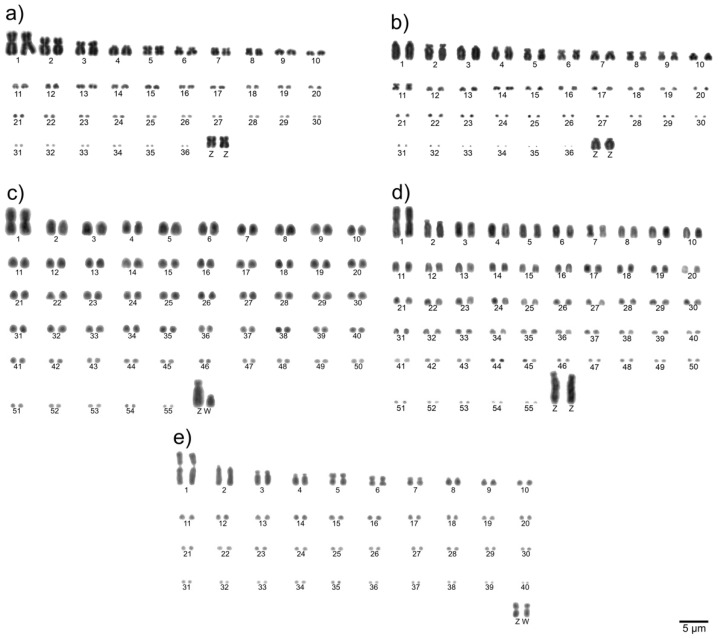
Complete Giemsa-stained karyotypes of Phalacrocorax brasilianus (**a**), Hydropsalis torquata (**b**), Pteroglossus inscriptus (**c**), Ramphastos tucanus tucanus (**d**), and Trogon surrucura surrucura (**e**).

The karyotype of *P*. *brasilianus* is 2*n* = 74 and is described here for the first time. The first, second, third and fifth pairs are submetacentric, sixth and eighth are metacentric, and the remaining autosomal pairs are acrocentric. The Z sex chromosome is submetacentric and equivalent in size to the third autosomal pair (Figure 1a). Corroborating previous studies, we found 2*n* = 74 in *H*. *torquata* [40], 2*n* = 112 in *P*. *inscriptus* and *R*. *t*. *tucanus* [11], and 2*n* = 82 in *T*. *s*. *surrucura* [32] (Figure 1b–e, respectively).

### 3.2. FISH Mapping Results and Reconstruction of the Neognathae/Neoaves Ancestors

**Figure 2:**
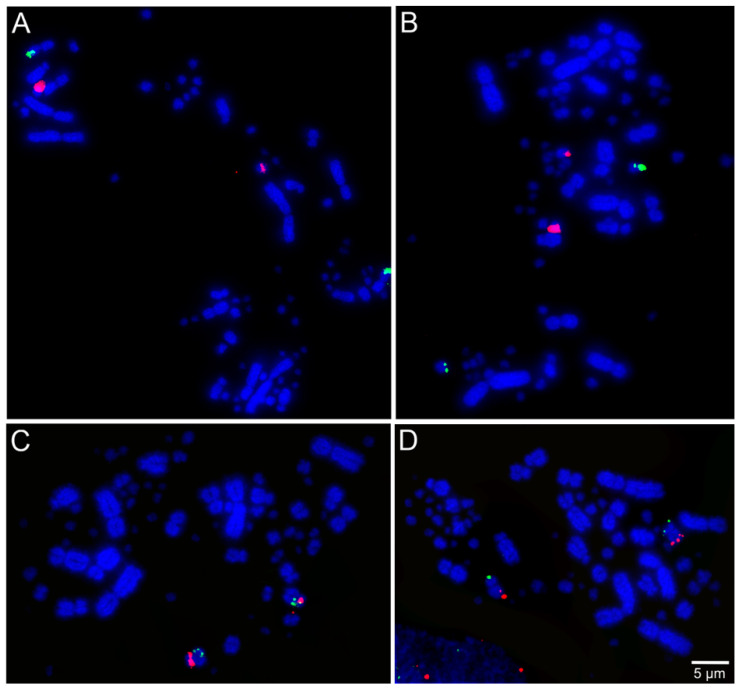
Examples of fluorescent in situ hybridization (FISH) experiments using chicken or zebra finch bacterial artificial chromosome (BAC) probes in *Phalacrocorax brasilianus*: (**A**) chicken macrochromosome 5 CH261-49B22 FITC and CH261-78F13 Texas red; (**B**) chicken macrochromosome 6 TGMCBA-382J4 FITC and CH261-49F3 Texas red; (**C**) chicken macrochromosome 9 CH261-183N19 FITC and chicken macrochromosome 10 CH261-115G24 Texas red; and (**D**) chicken microchromosome 11 CH261-154H1 FITC and chicken microchromosome 13 TGMCBA-321B13 Texas red.

**Figure 3:**
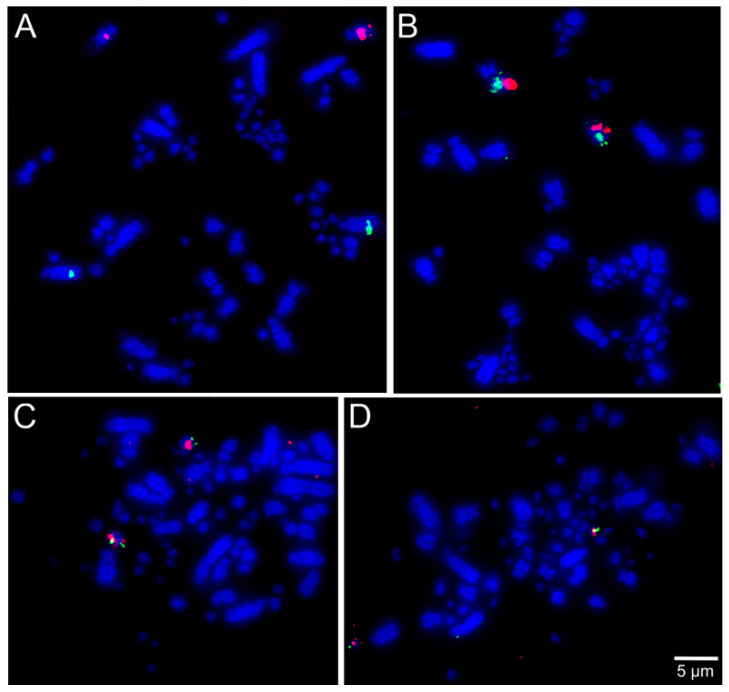
Examples of FISH experiments using chicken or zebra finch BAC probes in *Hydropsalis torquata*: (**A**) chicken macrochromosome 2 CH261-44D16 FITC and CH261-177K1 Texas red; (**B**) chicken macrochromosome 9 CH261-183N19 FITC and chicken microchromosome 13 TGMCBA-321B13 Texas red; (**C**) chicken microchromosome 12 CH261-60P3 FITC and CH261-4M5 Texas red; and (**D**) chicken microchromosome 27 CH261-66M16 FITC and CH261-28L10 Texas red.

**Figure 4:**
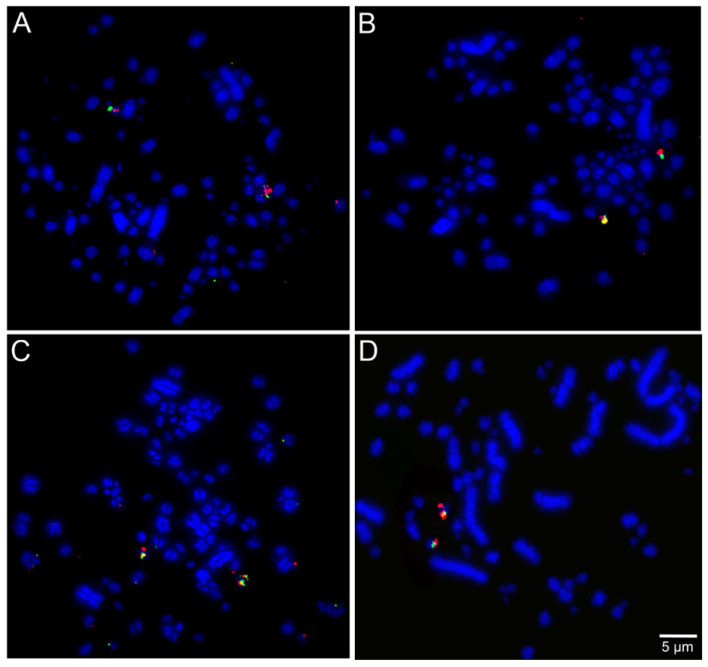
Examples of FISH experiments using chicken or zebra finch BAC probes in *Pteroglossus inscriptus*: (**A**) chicken microchromosome 19 CH261-50H12 FITC and CH261-10F1 Texas red; and (**B**) chicken microchromosome 22 CH261-40J9 FITC and CH261-18G17 Texas red). *Ramphastos tucanus tucanus*: (**C**) chicken microchromosome 17 CH261-42P16 FITC and TGMCBA-375I5 Texas red. *Trogon surrucura surrucura*: (**D**) chicken microchromosome 26 CH261-186M13 FITC and CH261-170L23 Texas red).

The selected BAC probes were successfully hybridized in all the five species included in our cross-species FISH mapping study, except the ones for chromosome pair 25 in *T*. *s*. *surrucura*. Examples of the respective FISH results are shown in Figure 2, Figure 3 and Figure 4.

**Figure 5:**
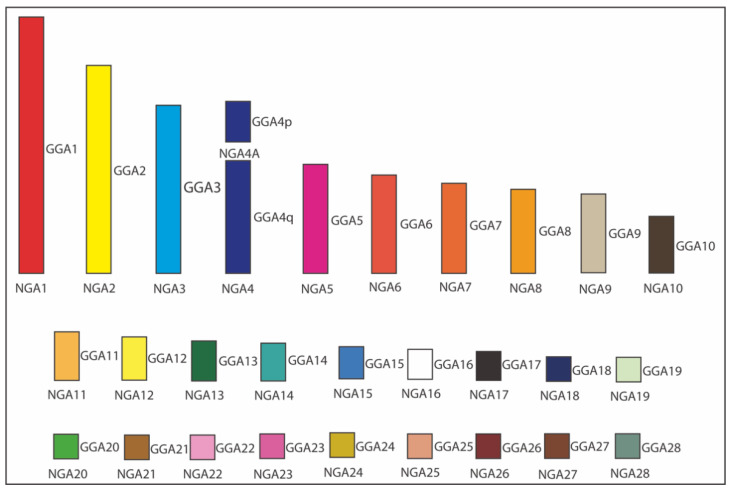
Ideogram of Neognathae ancestor (NGA) karyotype (NGA1 to NGA28). The NGA ancestral karyotype is likely to have the same homology with chicken (*Gallus gallus*; GGA), except GGA4 split into two separated chromosomes. Each GGA chromosome is illustrated with a different color. The white color indicates the probable homology with GGA16.

Using a web-based MLGO tool, the relevant input files for BAC order/orientation maps in the whole set of 12 species, and the relevant input phylogenetic tree (Figure S1), we performed an overall estimation of the hypothetical Neognathae (NGA) and Neoaves (NAA) ancestors (Figure 5). This was possible thanks to the chromosome maps for the ostrich (infraclass Palaeognathae) available from the O’Connor et al. [16] study and used as an outgroup. In the case of macrochromosomes, the orientation of each BAC relative to its neighbors on a particular chicken chromosome and relative to p/q arms was an additional advantage for the reconstruction of bird ancestors.

As a result of our ancestral karyotype reconstruction, a similar pattern of chromosome organization in the presumable NGA and NAA was observed (Figure 5). Overall, according to this gross estimate and using datasets for the 12 species produced in this and few other published studies, NGA and NAA are likely to have 29 chromosomes (autosomes), including 10 macrochromosomes (i.e., autosomes 1–9 + 4A) and 19 microchromosomes (i.e., autosomes 10–28). Compared to the chicken karyotype, the only difference between these two karyotypes and the chicken one was that chromosome GGA4 was split into two separate chromosomes (4 and 4A) in NGA and NAA. Considering that the infraclasses Palaeognathae (ratites and tinamous) and Neognathae (superorders Galloanserae and Neoaves) diverged about 100 million years ago (Mya) and the Neognathae diverged into the evolutionary lineages of Galloanserae and Neoaves about 88 Mya [41], we have used the most ancestral NGA karyotype to compare with our results obtained through the FISH experiments.

### 3.3. Rearrangements

**Figure 6:**
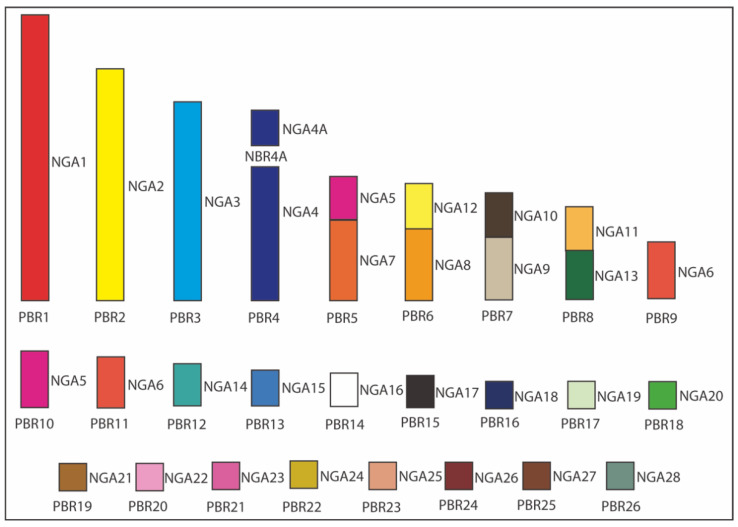
Homology map between *Phalacrocorax brasilianus* (PBR) and the Neognathae ancestral karyotype (NGA). NGA homologies are represented with a different color. The white color indicates the probable homology with NGA16.

The rearrangements (Table 2) were identified, comparing our results with the NGA karyotype. According to these comparisons, chromosome evolution in *P*. *brasilianus* involved macrochromosomal fissions, a fusion of macrochromosomes, a fusion between a macrochromosome and a microchromosome and fusions of microchromosomes. The chromosomes homologous to NGA5 and 6 were split into two pairs each in the karyotype of *P*. *brasilianus*. Four fusions overall were found: NGA5seg/7, NGA8/12, NGA9/10, and NGA11/13. Chromosomes NGA14–28 were conserved as discrete units in this species, following the ancestral pattern. Figure 6 shows the homology between the NGA karyotype and *P*. *brasilianus*.

**Figure 7:**
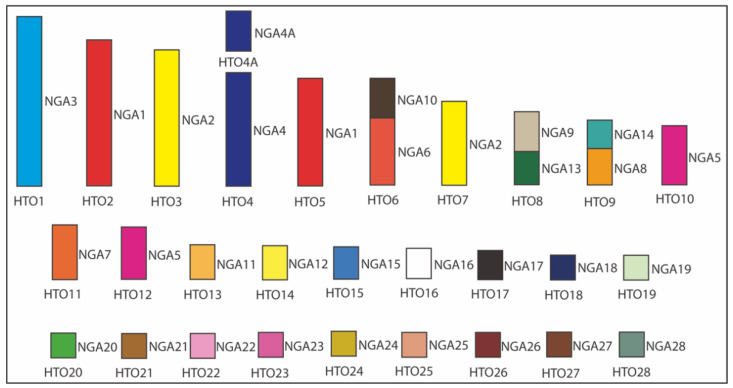
Homology map between *Hydropsalis torquata* (HTO) and the Neognathae ancestral karyotype (NGA). NGA homologies are represented with a different color. The white color indicates the probable homology with NGA16.

Fissions and fusions involving macro- and microchromosomes were also observed in *H*. *torquata*. Chromosomes homologous to NGA1, 2, and 5 are split into two pairs in the karyotype of *H*. *torquata*. Three associations were found: NGA6/10, NGA8/14, and NGA9/13. Chromosomes NGA11, 12, and 15–28 were conserved as respective microchromosomes in this species. Figure 7 shows the homology between the NGA karyotype and *H*. *torquata*.

Although a high diploid number is present in *P*. *inscriptus* and *R*. *t*. *tucanus* (2*n* = 112), no interchromosomal rearrangements involving the microchromosomes (NGA10–28) were found in these Piciformes species. Likewise, no interchromosomal rearrangements involving these microchromosomes were found in *T*. *s*. *surrucura* (2*n* = 82). Unlike the other species analyzed, no hybridization signs were produced when the BAC probes for chromosome 25 were applied to metaphase spreads of *T*. *s*. *surrucura*. The homology maps for these species are not shown because no interchromosomal rearrangements involving the microchromosomes were found, and the macrochromosome homology with *Gallus gallus* has been previously reported [11,32].

### 3.4. Comparison of Microchromosome Organization in Birds

We added our data to those previously published in the literature about chromosomal rearrangements in 18 microchromosomes in birds. In a total of 34 avian species (Supplementary Table S2), nine presented interchromosomal rearrangements (Table 3). The average frequency of species with interchromosomal rearrangements along the 18 microchromosomes was 10.3%, but we found significant heterogeneity among chromosomes (χ^2^ = 40.927, df = 17, *p* = 0.001). The frequency was higher in NGA10 (26%, *p* = 0.001), NGA13 (21%, *p* = 0.042) and NGA14 (23%, *p* = 0.009) (Table 3). No interchromosomal rearrangements were observed in microchromosomes NGA22, NGA24, NGA26 and NGA27 (all at *p* < 0.05). However, when adjusting for the number of tests performed, only in NGA10 the excess of species with rearrangements reached statistical significance (*p* = 0.025) (Table 3).

## 4. Discussion

In previous studies, BAC probes have been used for inter-cross FISH mapping to comprehend the structure and organization of the chromosomes in species from 12 orders (Table S2), and interchromosomal rearrangements were reported only in Falconiformes, Psittaciformes and Cuculiformes species [15,16,24,25,26,27,28] (Table S2). Here we cytogenomically analyzed five species from four Neoaves orders (Caprimulgiformes, Piciformes, Suliformes, and Trogoniformes), expanding the results to a total of 16 orders. Overall, the results demonstrated that interchromosomal rearrangements involving macro- and microchromosomes had an important role in the karyotype evolution of species of Caprimulgiformes and Suliformes, while the microchromosomes remained highly conserved in Piciformes and Trogoniformes. Our results suggest that the microchromosomes NGA10, NGA13 and NGA14 are involved in multiple rearrangements. However, only in NGA10, the frequency of rearrangements was supported statistically. In addition, this is the first molecular cytogenetic study in species of Suliformes and Caprimulgiformes.

### 4.1. Karyotype of P. brasilianus (Suliformes)

The karyotype of *P*. *brasilianus* (Suliformes) comprises 74 chromosomes and is reported here for the first time. Although this diploid number is slightly lower than the “typical” avian karyotype (2*n* ≈ 80), we detected fissions of chromosomes homologous to ancestral pairs 5 and 6 (NGA5 and NGA6). Chromosome fissions involving the homologous chromosome to NGA5 are frequent in birds and have been reported in species from the following Neoaves orders: Accipitriformes, Charadriiformes, Eurypygiformes, Falconiformes, Gruiformes, Passeriformes, Trogoniformes, Piciformes, and Strigiformes [7,30,42,43,44,45,46], while fissions in GGA6 have been detected previously only in Psittaciformes species [28,47,48] and in *Crotophaga ani*, a Cuculiformes species [27]. In addition, four chromosomal associations were detected in *P*. *brasilianus*, including associations between macrochromosomes (NGA5/7), macrochromosomes and microchromosomes (NGA8/12 and NGA9/10) and between microchromosomes (NGA11/13). Considering that the karyotype of *P*. *brasilianus* is similar to other Suliformes species, especially *Phalacrocorax bransfieldensis*, which shares the same diploid number [49], it is likely that microchromosome fusions are not exclusive to *P*. *brasilianus* in the order Suliformes.

### 4.2. Karyotype of H. torquata (Caprimulgiformes)

Fusion and fission events were also observed in *H*. *torquata* (Caprimulgiformes) (2*n* = 74). Chromosomal fissions were found in ancestral chromosome pairs 1, 2 and 5 (NGA1, 2 and 5). The breakpoints involved in these fissions are probably reused in bird chromosome evolution since fissions in these chromosomes were reported in several orders of birds [3,4,5,16]. However, the use of a higher number of BACs probes covering these chromosomes is necessary to confirm this hypothesis. In *H*. *torquata,* we found fusions between macrochromosomes (NGA6/10) and between macrochromosomes and microchromosomes (NGA9/13 and NGA8/14). Based on the fact that conventional cytogenetic analyses in Caprimulgiformes species revealed an interesting range of diploid number, from 2*n* = 68 in *Chordeiles pusillus* [50] to 2*n* = 86 in *Nyctibius griseus* [40], it is plausible to infer that fusions involving microchromosomes and macrochromosomes appear to have played an important role in the chromosome evolution of this group.

### 4.3. Karyotype of T. s. surrucura (Trogoniformes)

Chromosomal analysis of Trogoniformes species is still rare and is based only on the karyotype description and chromosome painting in *T*. *s*. *surrucura*, with 2*n* = 82 [32]. Although a microchromosome fusion was proposed in this species based on chicken macrochromosome painting [32], we did not find any evidence of this rearrangement in our analysis. However, given that there are no probes available for chicken chromosomes 16 and 29–38, we cannot entirely discard the occurrence of microchromosome fusions.

### 4.4. Karyotype of P. inscriptus and R. t. tucanus (Piciformes)

The diploid numbers found in *P*. *inscriptus* and *R*. *t*. *tucanus*, 2*n* = 112 in both species, raise questions concerning the rearrangements that may have led to this high diploid number. Comparative chromosome painting with chicken macrochromosome probes (GGA1–10, homologous to NGA1–10) has been performed, and extensive chromosomal fissions were found in the first five ancestral chromosome pairs (NGA1–5) [11]. However, the results presented here demonstrate the conservation of the ancestral patterns of microchromosomes in both species, suggesting that the fission events exclusively involved macrochromosomes. This finding suggests that the high diploid number observed in Ramphastidae species is a result of macrochromosomal fission only. Another interesting feature observed in Piciformes species is that the Z sex chromosome is the largest element of the karyotype [11,51]. Our study excludes the possibility that a fusion event took place between the Z chromosome and any of the microchromosomes tested here, corroborating a previous hypothesis that fissions of the macrochromosomes and the accumulation of repetitive sequences are the most likely mechanism responsible for the appearance of this enlarged sex chromosome in Piciformes species [11].

### 4.5. Overview of Interchromosomal Rearrangements Involving Microchromosomes in Birds

So far, no patterns of interchromosomal rearrangement have been reported as being shared among the species that exhibit rearrangements involving microchromosomes, e.g., from Cuculiformes [27], Psittaciformes [16], Falconiformes [15,25], Caprimulgiformes, and Suliformes (present study; Table S2). However, while microchromosomal fusions were shared by three Falconiformes species [15,25], each of the four Psittaciformes species studied exhibited a different pattern of microchromosomal fusions [15]. This would suggest that the convergent evolution of microchromosomal rearrangements seems to be a rare event in birds and may be an appropriate tool for phylogenetic analyses in the taxa where these rearrangements are present.

While it is evident that there are karyotypes highly rearranged and interchromosomal rearrangements involving microchromosomes were expected in Cuculiformes, Psittaciformes and Falconiformes [15,16,25,27,28], such rearrangements were not evident in the Giemsa-staining karyotypes of *P*. *brasilianus* and *H*. *torquata*, despite the fact that their diploid numbers are slightly lower than the putative avian ancestral karyotype (2*n* = 80). Hence, even in species with conserved karyotypes at first glance, microchromosomal fusions may have played an important role in their karyotype evolution.

Some microchromosome syntenies are involved in multiple rearrangements; for example, the frequency of species with interchromosomal rearrangements was higher in three syntenic groups, homologous to microchromosomes NGA10, NGA13 and NGA14 (Table 2). However, only in NGA10, the frequency of rearrangements was statistically higher than the average (Table 2). Why this microchromosome is more prone to interchromosomal rearrangement than others remains unclear and deserves futures studies. We did not observe rearrangements in pairs NGA22, 24, 26 and 27, but this result was not supported by the statistical analysis. A possible explanation could be the small sample size studied (34 species), but the real stability of these microchromosomes to rearrangements cannot be ruled out until new studies are done.

Our results corroborate the recent suggestion that microchromosomes are not prone to breakage [15] since no fissions in these elements were observed to date. However, it is unclear why interchromosomal rearrangements involving microchromosomes are quite common in some orders (e.g., Falconiformes, Psittaciformes, Caprimulgiformes, Cuculiformes, and Suliformes), while in most other avian orders, they have remained largely unchanged [15]. Nevertheless, as more chromosome mapping of BAC probes is performed, the list of orders with microchromosome fusions is likely to increase, with some species-specific rearrangements being detected. Future cytogenomic studies using this approach will provide greater clarity on why microchromosomes remain conserved as discrete units in some species while they are prone to interchromosomal rearrangement in others.

Concluding, in this cross-species FISH mapping study, we have reported and characterized the organization of microchromosomes in species from four different Neoaves orders. The results have further contributed to avian cytogenomics, revealing that microchromosome fusions are not exclusive to the orders Cuculiformes, Falconiformes and Psittaciformes but are also inherent in representatives of the orders Caprimulgiformes and Suliformes. Additionally, our findings suggested that some microchromosomes are more likely to undergo interchromosomal rearrangements than others and that convergent evolution of microchromosomal rearrangements is a rare event in birds.

## Figures and Tables

**Table 1:** List of the avian Neoaves species analyzed and the approaches used.

Order	Species Name	Sex	Locality/ State *	Macrochromosome Information	Microchromosome Information
Caprimulgiformes	*Hydropsalis torquata*	Male	Porto Vera Cruz/RS	Present study	Present study
Piciformes	*Pteroglossus inscriptus*	Female	Parque Zoobotânico do Museu Paraense Emilio Goeldi/PA	[11]	Present study
Piciformes	*Ramphastos tucanus*	Male	Parque Zoobotânico do Museu Paraense Emilio Goeldi/PA	[11]	Present study
Suliformes	*Phalacrocorax brasilianus*	Male	Parque Mangal das Garças/PA	Present study	Present study
Trogoniformes	*Trogon surrucura surrucura*	Female	Porto Vera Cruz/RS	[32]	Present study

* Brazilian states: RS, Rio Grande do Sul; PA, Pará.

**Table 2:** Patterns of interchromosomal rearrangements revealed in the karyotype of five species of Neoaves birds.

NGA Chromosomes	*P. brasilianus*	*H*. *torquata*	*P*. *inscriptus*	*R*. *t*. *tucanus*	*T*. *s*. *surrucura*
NGA1	–	Fissions in two pairs	Fission in six pairs; NGA1seg fused with NGA3seg	Fission in six pairs; NGA1seg fused with NGA3seg	–
NGA2	–	Fissions in two pairs	Fission in six pairs	Fission in six pairs	Fission in two pairs
NGA3	–	–	Fission in six pairs; NGA3seg fused with NGA1seg	Fission in six pairs; NGA3seg fused with NGA1seg	–
NGA4q	–	–	Fission in two pairs	Fission in two pairs	–
NGA4p	–	–	–	–	–
NGA5	Fissions in two pairs; NGA5seg fused with NGA7	Fissions in two pairs	Fission in three pairs	Fission in three pairs	–
NGA6	Fissions in two pairs	Fusion with NGA10	–	–	Fusion with NGA7
NGA7	Fusion with NGA5seg	–	–	–	Fusion with NGA6
NGA8	Fusion with NGA12	Fusion with NGA14	–	–	–
NGA9	Fusion with NGA10	Fusion with NGA13			–
NGA10	Fusion with NGA9	Fusion with NGA6	–	–	–
NGA11	Fusion with NGA13	–	–	–	–
NGA12	Fusion with NGA8	–	–	–	–
NGA13	Fusion with NGA11	Fusion with NGA9	–	–	–
NGA14	–	Fusion with NGA8	–	–	–
NGA15	–	–	–	–	–
NGA16	No data	No data	No data	No data	No data
NGA17	–	–	–	–	–
NGA18	–	–	–	–	–
NGA19	–	–	–	–	–
NGA20	–	–	–	–	–
NGA21	–	–	–	–	–
NGA22	–	–	–	–	–
NGA23	–	–	–	–	–
NGA24	–	–	–	–	–
NGA25	–	–	–	–	No data
NGA26	–	–	–	–	–
NGA27	–	–	–	–	–
NGA28	–	–	–	–	–

The chromosomal correspondence to NGA1–10 of *P*. *inscriptus* and *R*. *t*. *tucanus* from Kretschmer et al. [11] and *T*. *s*. *surrucura* from Degrandi et al. [32]. NGA = Neognathae ancestral karyotype; seg = segment.

**Table 3:** Number of avian species with and without interchromosomal rearrangements in 18 microchromosomes, considering 34 avian species, which had been already studied with BAC probes for microchromosomes.

Microchromosome	Interchromosomal Rearrangement		With %	Residual Analysis		*p* Values	Adjusted *p* Values
	With	Without		With	Without		
NGA10	9	25	**26.5**	**3.19**	**−3.19**	**0.0014**	**0.0252**
NGA11	5	29	14.7	0.87	−0.87	0.384	0.6912
NGA12	6	28	17.6	1.45	−1.45	0.147	0.2940
NGA13	7	27	**20.6**	**2.03**	**−2.03**	**0.042**	0.1080
NGA14	8	26	**23.5**	**2.61**	**−2.61**	**0.009**	0.0810
NGA15	4	30	11.8	0.29	−0.29	0.772	0.7720
NGA17	4	30	11.8	0.29	−0.29	0.772	0.7720
NGA18	3	31	8.8	−0.29	0.29	0.772	0.7720
NGA19	3	31	8.8	−0.29	0.29	0.772	0.7720
NGA20	4	30	11.8	0.29	−0.29	0.772	0.7720
NGA21	3	31	8.8	−0.29	0.29	0.772	0.7720
NGA22	0	34	**0.0**	−2.03	2.03	**0.042**	0.1080
NGA23	3	31	8.8	−0.29	0.29	0.772	0.7720
NGA24	0	34	**0.0**	**−2.03**	**2.03**	**0.042**	0.1080
NGA25	1	33	2.9	−1.45	1.45	0.147	0.2940
NGA26	0	34	**0.0**	**−2.03**	**2.03**	**0.042**	0.1080
NGA27	0	34	**0.0**	**−2.03**	**2.03**	**0.042**	0.1080
NGA28	3	31	8.8	−0.29	0.29	0.772	0.7720

Heterogeneity among microchromosomes chi-squared = 40,927, df = 17, exact *p*-value = 0.001. Statistically significant values are shown in bold.

## Data Availability

All the data supporting our findings are contained within the manuscript.

## Supplementary Materials

The following are available online at https://www.mdpi.com/article/10.3390/cells10040826/s1, Figure S1: The MLGO input phylogenetic tree, Table S1: Identification of BAC probes used in this study, Table S2: List of avian species analyzed with microchromosome BAC probes.
