# Supplementary material for: Interspecies Chromosome Mapping in Caprimulgiformes, Piciformes, Suliformes, and Trogoniformes (Aves): Cytogenomic Insight into Microchromosome Organization and Karyotype Evolution in Birds
Source: Cells. 2021 Apr 7;10(4):826. doi: 10.3390/cells10040826 (PMC8067558; doi:10.3390/cells10040826)
Supplement: Supplementary file 1 [file cells-10-00826-s001.zip › Figure S1.docx]

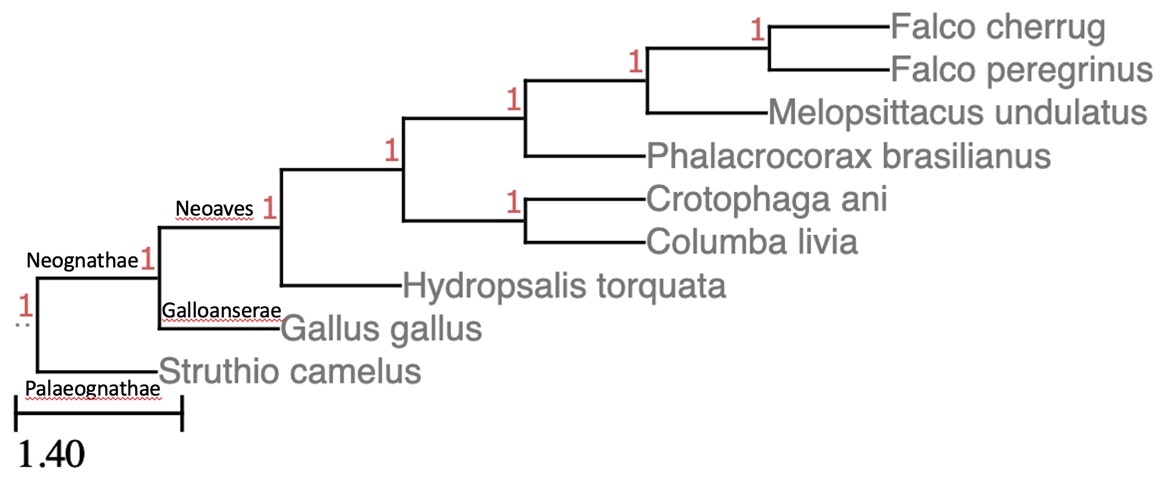


Figure S1. The MLGO input phylogenetic tree. The tree includes 11 birds plus ostrich taken as an outgroup genome and was visualised using the ETE v3 toolkit (Hu et al., 2014). The respective Newick format tree can be written as (((((((Falco cherrug,Falco peregrinus),Melopsittacus undulatus),Phalacrocorax brasilianus),(Crotophaga ani,Columba livia)),Hydropsalis torquata),Gallus gallus),Struthio camelus); as inferred from the Prum et al. (2015) phylogeny for birds. Provisional support values (1) are shown in red.
