# Supplementary material for: Interspecies Chromosome Mapping in Caprimulgiformes, Piciformes, Suliformes, and Trogoniformes (Aves): Cytogenomic Insight into Microchromosome Organization and Karyotype Evolution in Birds
Source: Cells. 2021 Apr 7;10(4):826. doi: 10.3390/cells10040826 (PMC8067558; doi:10.3390/cells10040826)
Supplement: Supplementary file 1 [file cells-10-00826-s001.zip › Table S2.docx]

Table S1 - List of avian species analyzed with microchromosome BAC probes.

| **Species** | **Order** | **Rearrangement involving microchromosomes** | **Reference** |
| --- | --- | --- | --- |
| *Pteroglossus inscriptus* | Piciformes | No | Present study |
| *Ramphastos tucanus tucanus* | Piciformes | No | Present study |
| *Trogon surrucura surrucura* | Trogoniformes | No | Present study |
| *Nannopterum brasilianus* | Suliformes | NGA7/5;  NGA12/8;  NGA10/9;  GGA13/11 | Present study |
| *Hydropsalis torquata* | *Caprimulgiformes* | NGA10/6;  NGA13/9;  NGA14/8 | Present study |
| *Gallinula melanops* | Gruiformes | No | [29] |
| *Crotophaga ani* | Cuculiformes | NGA5/15  NGA11/8  NGA4A/12  NGA25/10  NGA14/6  NGA4/13  NGAZ/17 | [27] |
| *Columbina passerina* | Columbiformes | No | [26] |
| *Columbina talpacoti* | Columbiformes | No | [26] |
| *Patagioenas cayennensis* | Columbiformes | No | [26] |
| *Geotrygon violacea* | Columbiformes | No | [26] |
| *Geotrygon montana* | Columbiformes | No | [26] |
| *Anas platyrhynchos* | Anseriformes | No | [15] |
| *Scolopax rusticola* | Charadriiformes | No | [15] |
| *Columba livia* | Columbiformes | No | [15] |
| *Streptopelia decaocto* | Columbiformes | No | [15] |
| *Falco peregrinus* | Falconiformes | NGA6/17/5/10;  NGA4/15/19/18;  NGA2/21/23;  NGA2/28/14/12;  NGA7/13;  NGA5/20 | [15] |
| *Falco cherrug* | Falconiformes | NGA4/15/19/18;  NGA2/21/23;  NGA2/28/14/12;  NGA5/10;  NGA7/13;  NGA6/17;  NGA5/20 | [15] |
| *Falco rusticolus* | Falconiformes | NGA4/15/19/18;  NGA2/21/23;  NGA2/28/14/12;  NGA5/10;  NGA7/13;  NGA6/17;  NGA5/20 | [15] |
| *Meleagris gallopavo* | Galliformes | No | [15] |
| *Coturnix chinensis* | Galliformes | No | [15] |
| *Coturnix japonica* | Galliformes | No | [15] |
| *Numida meleagris* | Galliformes | No | [15] |
| *Pavo cristatus* | Galliformes | No | [15] |
| *Ammoperdix heyi* | Galliformes | No | [15] |
| *Chlamydotis undulata* | Otidiformes | No | [15] |
| *Turdus merula* | Passeriformes | No | [15] |
| *Serinus canaria* | Passeriformes | No | [15] |
| *Taeniopygia guttata* | Passeriformes | No | [15] |
| *Melopsittacus undulatus* | Psittaciformes | NGA3/17/3;  NGA5/6/7/6;  NGA8/9/4A;  NGA4/11;  NGA14/5/7;  NGA12/10;  NGA20/13 | [15] |
| *Nymphicus hollandicus* | Psittaciformes | NGA10/seg;  NGA11/1;  NGA14/seg; | [15] |
| *Cyanoramphus novaezelandiae* | Psittaciformes | NGA10/seg;  NGA11/seg;  NGA14/seg; | [15] |
| *Bubo ascalaphus* | Strigiformes | No | [15] |
| *Struthio camelus* | Struthioniformes | No | [15] |

Although fusions involving microchromosomes were detected in *Myiopsitta monachus*, these data were not compared here because the exact fusions involving chromosomes GGA10, 11, and 12 were not identified, since a flow sorted peak including the chromosomes GGA10, 11, and 12 was used [28]. Likewise, the data of *Willisornis vidua* were not compared here because the authors reported several interchromosomal rearrangements in this species, however, the exact rearrangements were not described [30].
